# Isolation, Virulence, and Antimicrobial Resistance of Methicillin-Resistant *Staphylococcus aureus* (MRSA) and Methicillin Sensitive *Staphylococcus aureus* (MSSA) Strains from Oklahoma Retail Poultry Meats

**DOI:** 10.3390/ijerph120606148

**Published:** 2015-05-29

**Authors:** Lubna S. Abdalrahman, Adriana Stanley, Harrington Wells, Mohamed K. Fakhr

**Affiliations:** Department of Biological Science, The University of Tulsa, Tulsa, OK 74104, USA; E-Mails: Lubna-abdalrahman@utulsa.edu (L.S.A.); Adriana-Stanley@utulsa.edu (A.S.); Harrington-wells@utulsa.edu (H.W.)

**Keywords:** *Staphylococcus aureus*, MRSA, antibiotic resistance, toxin genes, organic, foodborne pathogens, retail poultry, spa, MLST, PFGE

## Abstract

*Staphylococcus aureus* is one the top five pathogens causing domestically acquired foodborne illness in the U.S. Only a few studies are available related to the prevalence of *S. aureus* and MRSA in the U.S. retail poultry industry. The objectives of this study were to determine the prevalence of *S. aureus* (MSSA and MRSA) in retail chicken and turkey meats sold in Tulsa, Oklahoma and to characterize the recovered strains for their antimicrobial resistance and possession of toxin genes. A total of 167 (114 chicken and 53 turkey) retail poultry samples were used in this study. The chicken samples included 61 organic samples while the rest of the poultry samples were conventional. The overall prevalence of *S. aureus* was 57/106 (53.8%) in the conventional poultry samples and 25/61 (41%) in the organic ones. Prevalence in the turkey samples (64.2%) was higher than in the chicken ones (42.1%). Prevalence of *S. aureus* did not vary much between conventional (43.4%) and organic chicken samples (41%). Two chicken samples 2/114 (1.8%) were positive for MRSA. PFGE identified the two MRSA isolates as belonging to PFGE type USA300 (from conventional chicken) and USA 500 (from organic chicken) which are community acquired CA-MRSA suggesting a human based source of contamination. MLST and spa typing also supported this conclusion. A total of 168 *Staphylococcus aureus* isolates (101 chicken isolates and 67 turkey isolates) were screened for their antimicrobial susceptibility against 16 antimicrobials and their possession of 18 different toxin genes. Multidrug resistance was higher in the turkey isolates compared to the chicken ones and the percentage of resistance to most of the antimicrobials tested was also higher among the turkey isolates. The hemolysin *hla* and *hld* genes, enterotoxins *seg* and *sei,* and leucocidins *lukE-lukD* were more prevalent in the chicken isolates. The PVL gene *lukS-lukF* was detected only in chicken isolates including the MRSA ones. In conclusion, *S. aureus* is highly prevalent in poultry retail meats sold in Oklahoma with a very low presence of human-originated MRSA. Multidrug resistance is not only prevalent in the MRSA isolates, but also in many MSSA poultry strains, particularly turkey.

## 1. Introduction

*Staphylococcus aureus,* with an estimated 241,148 cases annually, is listed among the top five pathogens causing domestically acquired foodborne illness in the U.S. [1]. Methicillin Resistant *S. aureus* (MRSA) acquires resistance to penicillin and other β-lactam antibiotics by harboring the *mecA* gene encoding the penicillin binding protein 2a (PBP2a) [2]. The presence of *S. aureus* and MRSA in retail meat has recently drawn some attention. Contamination of retail meats by *Staphylococcus aureus* may occur as a result of poor food safety practices during handling the meat or directly from infected food-producing animals [3,4]. *S. aureus* and MRSA were isolated from cattle, pigs, and chickens, where a total of 421 samples were *S. aureus* and 15 were MRSA [5]. In Louisiana, a study found 45.6% of pork samples contained *S. aureus*, as well as 20% of beef samples, while MRSA was found in 5.6% of pork samples and 3.3% of beef samples [4]. *S. aureus* was also detected in lamb samples and chicken parts [6]. *S. aureus* and MRSA have been also isolated from retail chicken meat in Japan, where two isolates of MRSA were detected out of 444 samples of raw chicken meats purchased from 145 different supermarkets [7] and the highest prevalence of MRSA in poultry was in The Netherlands where MRSA was prevalent in 35.3% of retail turkey meat samples [8].

While some studies were conducted to determine the prevalence of *S. aureus* and MRSA in retail meats sold in the U.S., including Louisiana, Maryland, Michigan, Iowa, Minnesota, New Jersey, Georgia, and North Dakota [4,9,10,11,12,13,14,15], only a few of those studies included data from poultry. *S. aureus* was detected in 56% of ground turkey where 17% of the isolated strains were resistant to methicillin, but no MRSA was detected in Maryland [9]. Twenty five percent of chicken and turkey retail samples were positive for *S. aureus*, while 3.9% of chicken and 1.7% of turkey samples were positive for MRSA in Michigan [10]. In a study in Iowa, the prevalence of *S. aureus* was 19.4% in turkey and 17.8% in chicken, but no MRSA was isolated from their poultry meat samples [11]. A higher prevalence of *S. aureus* (67.6%) was detected in chicken meats in North Dakota [14]. While most studies conducted in Europe identified MRSA isolated from retail meats as belonging to livestock- acquired MRSA strains (LA-MRSA) [8], the majority of MRSA isolates detected in the U.S. retail meats were human associated strains [4,10], except a few that were LA-MRSA and mostly reported in pork meat [14,15].

As mentioned above, the number of studies discussing the prevalence of *S. aureus* in U.S. retail poultry meats is limited in the literature. The objectives of this study were to determine the prevalence of *Staphylococcus aureus* and MRSA in retail chicken and turkey meats sold in the Tulsa (Oklahoma) area and to characterize the recovered strains for their antimicrobial resistance and possession of toxin genes.

## 2. Experimental Section

### 2.1. Isolation of Staphylococcus aureus and MRSA from Retail Chicken and Turkey

Retail poultry samples were collected from several grocery stores in Tulsa, Oklahoma on a weekly base from January to June 2010. A total of 167 (114 chicken and 53 turkey) chilled retail poultry samples were used in this study. While 61/114 of the chicken samples were organic, the rest of the chicken samples (53/114) were conventional. All of the 53 turkey samples were conventional. Samples were selected to be as variable as possible with different brands, expiration dates, production codes, and cuts (chicken breast, chicken leg quarters, chicken drumsticks, chicken thighs, ground chicken, turkey drumsticks, turkey breast, turkey neck pieces, and ground turkey). Isolation of MSSA and MRSA was conducted as described previously [4,16,17]. Four to six suspected *S. aureus* strains out of each positive poultry sample were subjected to molecular identification.

### 2.2. DNA Extraction and Identification by PCR

DNA was extracted from the prospective *S. aureus* isolates using the single cell lysing buffer (SCLB) method [18] as described previously [16,19]. A multiplex PCR reaction was used to identify the suspected *S. aureus* isolates by using specific primers for *S. aureus* and MRSA to amplify a 108 bp and a 312 bp fragments, respectively [19]*.* Isolates that were negative for the *mecA* gene and showed resistance to cefoxitin and/or oxacillin were subjected to PCR confirmation using a second set of MRSA primers that amplify a 533 bp *mecA* fragment and two other *mecC* primer sets that amplify 356 bp and 1800 bp fragments to confirm the MRSA phenotype. All primers used were published elsewhere [20,21,22,23,24].

### 2.3. Molecular Typing of MRSA and MSSA Isolates

Twelve recovered MRSA isolates were subjected to PFGE according to the CDC protocol as described previously [25]. Plugs digested with *smaI* were run in a Seakem agarose gel (Lonza, Allendale, NJ, USA) with 0.5 × Tris-Borate EDTA (TBE) buffer (Amresco, Solon, OH, USA) on the CHEF Mapper PFGE system (Bio-Rad, Hercules, CA, USA) by running it for 18 h at 14 °C with initial switch time of 5 s, final switch time for 40 s. *Salmonella enterica* serovar Braenderup digested with *XbaI* (Promega, Madison, WI, USA) was used as the molecular reference marker. Gels were stained with ethidium bromide and viewed and recorded under UV transillumination (UVP, Upland, CA, USA). Gel images were analyzed using the BioNumerics software (Applied Maths, Austin, TX, USA). The MRSA isolates were also subjected to spa typing and MLST according to published primers and protocols. A subset of the recovered MSSA isolates was subjected to molecular typing using spa typing. Spa types were assigned using the BioNumerics Softwatre (Applied Math, Austin, TX, USA).

### 2.4. Antimicrobial Resistance Screening of Staphylococcus aureus and MRSA Isolates

A total of 168 *Staphylococcus aureus* isolates (101 chicken isolates and 67 turkey isolates) and 12 MRSA isolates (6 representatives of each of the two positive MRSA chicken samples) were subjected to antimicrobial resistance profiling against sixteen different antimicrobials that belong to ten different antibiotic classes (Table 1). Antimicrobial susceptibility testing was performed as described previously [16,17] Breakpoints established for each antimicrobial were used according to the Clinical and Laboratory Standards Institute (CLSI) when available [26].

**Table 1:** A list of the sixteen tested antimicrobials, their classes, and the concentrations used for susceptibility testing.

Antimicrobial Class	Antimicrobial	MIC Range (μg/mL)
β-lactams	penicillin	0.125–1
	ampicillin	0.25–2
	oxacillin + 2% NaCl	2–16
	cefoxitin + 2% NaCl	4–32
Tetracyclines	tetracycline	8–64
	doxycycline	8–64
Macrolides	azithromycin	4–32
	erythromycin	4–32
Aminoglycosides	kanamycin	32–256
	gentamicin	8–64
Fluoroquinolones	ciprofloxacin	2–16
Lincosamides	clindamycin	2–16
Phenicols	chloramphenicol	16–128
Glycopeptides	vancomycin	16–128
Rifamycines	rifampin	2–16
Sulfonamides	trimethoprim/sulfamethoxazole	2/38–16/304

### 2.5. Prevalence of Toxin Genes of Staphylococcus aureus and MRSA Isolates

A total of 168 *Staphylococcus aureus* isolates (101 chicken isolates and 67 turkey isolates) and 12 MRSA isolates (six representatives of each of the two positive MRSA chicken samples) were screened for eighteen different toxin genes that belong to six different toxin gene groups that include enterotoxins (*sea*, *seb-sec*, *sec*, *sed*, *see*, *seg*, *seh*, *sei*, *sej*), toxic shock syndrome toxin 1 (*tst*), exfoliative toxins (*eta*, *atb*), leucocidins (*lukE-lukD*, *lukM*), Panton-Valentine leucocidin (PVL) (*lukS-lukF*), and hemolysins (*hla, hlb, hld*). Primers used were published elsewhere [27,28,29]. Three multiplex reactions each of which included six toxin genes were performed as explained previously [16,17]. Several representative amplicons of each positive toxin were sequenced in house using the same amplifying primers to confirm PCR accuracy.

## 3. Results and Discussion

### 3.1. Prevalence of Staphylococcus aureus and MRSA in Chicken and Turkey

A total of 167 (114 chicken and 53 turkey) chilled retail poultry samples were used in this study (Table 2). While 61/114 of the chicken samples were organic, the rest of the chicken samples (53/114) were conventional. All of the 53 turkey samples were conventional. As shown in Table 2, the overall prevalence of *S. aureus* in the conventional poultry samples was 57/106 (53.8%) and in the organic poultry samples was 25/61 (41%). Prevalence of *S. aureus* in the turkey samples (64.2%) was higher than its prevalence in chicken samples (42.1%). Also the prevalence of *S. aureus* in the conventional turkey (64.2%) was at a higher percentage than in the conventional chicken (43.4%). Two samples out of 114 (1.8%) in the chicken were positive for MRSA*.* The overall prevalence of MRSA in the conventional poultry samples was 1/106 (0.9%) and in the organic poultry samples was 1/61 (1.6%) (Table 2). One MRSA strain was recovered from the organic chicken samples and the other one from a conventional chicken sample. None of the turkey samples were positive for MRSA*.*

**Table 2:** Prevalence of *Staphylococcus aureus* and MRSA in the collected chicken and turkey samples in relation to their conventional or organic sources.

Prevalence of *Staphylococcus aureus*									
	Chicken			Turkey			Poultry		
	Conventional * np/n (%)	Organic np/n (%)	Total np/n (%)	Conventional np/n (%)	Organic np/n (%)	Total np/n (%)	Conventional np/n (%)	Organic np/n (%)	Total np/n (%)
MSSA	23/53	25/61	48/114	34/53	0/0	34/53	57/106	25/61	82/167
	(43.4)	(41)	(42.1)	(64.2)	(0)	(64.2)	(53.8)	(41)	(49.1)
MRSA	1/53	01/61	02/114	0/53	0/0	0/53	1/106	1/61	02/167
	(1.9)	(1.6)	(1.8)	(0)	(0)	(0)	(0.9)	(1.6)	(1.2)

* np: Number of positive samples, n: Number of samples collected.

A chi Square test of independence was used to determine whether statistical differences in *S. aureus* prevalence occurred between organic and conventional chicken, and then between chicken and turkey. The prevalence of *S. aureus* did not differ significantly between conventional and organic chicken (*X*^2^ = 0.067, df = 1, *p* > 0.75). Since no significant difference occurred between organic and conventional chicken, the groups were combined for further analysis. The prevalence of *S. aureus* was significantly different between chicken and turkey (*X*^2^ = 7.036, df = 1, *p* < 0.01), with turkey having the higher prevalence. The same analysis was performed to test for significant difference in the prevalence of MRSA among sample groups. No significant difference in MRSA prevalence was found between conventional and organic chicken (*X*^2^ = 0.010, df = 1, *p* > 0.90), or between chicken and turkey (*X*^2^ = 0.941, df = 1, *p* > 0.25).

The percentage of chicken samples contaminated with *S. aureus* in our study was 42.1% (Table 2) and almost in agreement with a previous U.S. study (41%) [30]*.* This contamination rate is lower than that observed in a survey previously conducted in Japan that revealed a prevalence of 65.8% *S. aureus* in chicken meat [7] and another study in North Dakota where it was 67.6% [14]. It is also lower than that of Gundoga *et al.* who reported 53.3% of *S. aureus* in chicken meat [6]. The prevalence rate of 42.2% of *S. aureus* in chicken in our study was higher than a study in Michigan, where it was only 25% [10], and another study in Iowa where it was only 17.8% [11]. In the current study the overall prevalence of *S. aureus* in turkey was 64.2% (Table 2), which was slightly lower than another study where 77% of turkey was positive for *S. aureus* [30], and slightly higher than its prevalence in ground turkey in a U.S. study where it was 56% [9]. A study in Iowa reported a lower prevalence of *S. aureus* in turkey at 19.4% [11].

In our study, two chicken meat samples were positive for *mecA*-containing MRSA at a prevalence of 2/114 (1.8%) (Table 2). In a study in Japan 2/444 chicken samples were positive for MRSA [7] and in a U.S. study in Michigan, 3.9% of chicken samples were positive for MRSA [10]. No MRSA was detected in retail turkey in our study but in the same Michigan study mentioned above, MRSA was detected in 1.7% of the turkey meat samples [10]. No MRSA was detected in turkey meats in two other studies conducted in Maryland and Iowa [9,11].

**Figure 1:**
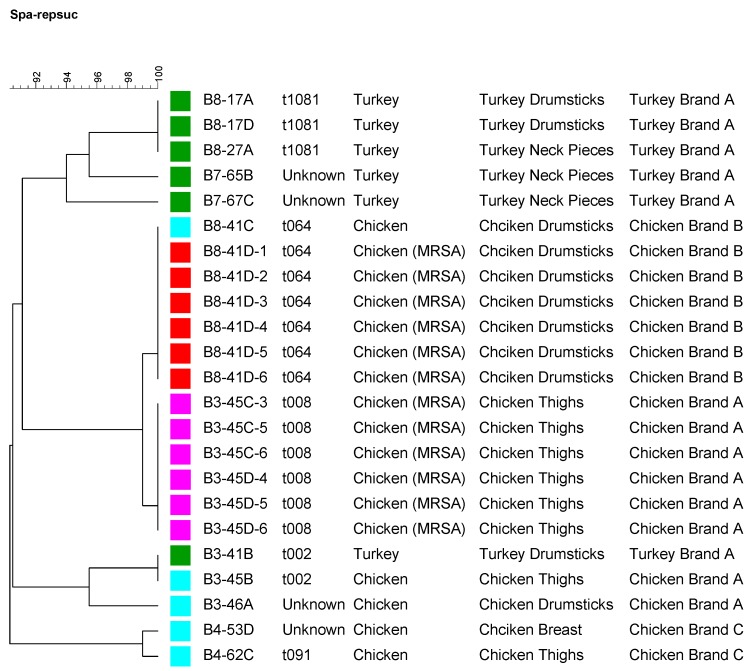
A dendrogram showing *spa* typing for a subset of the recovered *Staphylococcus aureus* strains including MRSA representing different meat sources, cuts, and brands. Strains isolated from the same meat source are labeled by the same color square.

**Figure 2:**
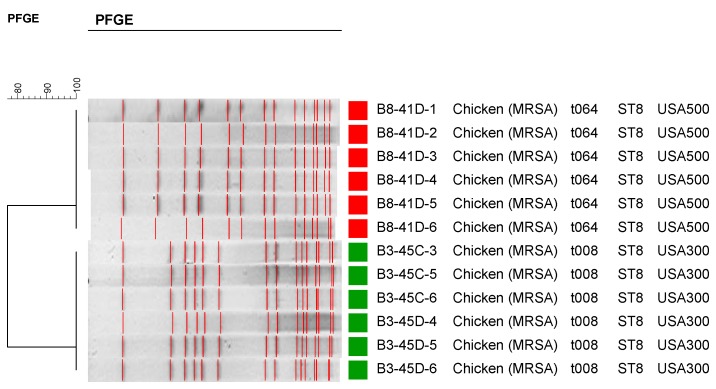
Pulsed Field Gel Electrophoresis (PFGE) patterns, spa types, and MLST of the twelve recovered MRSA strains.

A subset of the MSSA isolates recovered in this study was subjected to spa typing (Figure 1). As shown in Figure 1, most of the turkey isolates were clustered together. Different brands also appeared to cluster together according to their spa repeats. Twelve MRSA isolates representing the two positive MRSA chicken samples (6 MRSA isolates were recovered out of each positive chicken samples) were subjected to PFGE, spa typing and MLST (Figure 2). The first six MRSA isolates that came from a conventional chicken sample belonged to PFGE type USA300 (data not shown), which is a community acquired CA-MRSA. The second six MRSA isolates recovered from an organic chicken sample belonged to PFGE type USA500 (Figure 2), which is also a community acquired MRSA. MLST and spa typing also showed t046, t008, and ST08 which are consistent with the PFGE results indicating a human origin rather than livestock association. These results affirm that the source of MRSA contamination in the two chicken samples in our study is human based and may be due to improper handling of the meat at the slaughter house, processing facilities, or at the retail stores. This is not surprising since the majority of MRSA isolates detected in the US retail meats were human associated strains [4,10] except for few that were LA-MRSA and were mostly reported in pork meat [11,14,15], while most studies conducted in Europe identified MRSA from retail meats as Live Stock acquired MRSA strains (LA-MRSA) including pork, beef and turkey [8]. For example, all MRSA isolated from Michigan retail meats were USA300 [10]. Pu *et al.* also reported similar results where their MRSA strains belonged to USA100 and USA300, which are human HA-MRSA and CA-MRSA, respectively [4].

### 3.2. Antimicrobial Susceptibility of the Recovered Isolates

A total of 168 *Staphylococcus aureus* isolates (101 chicken isolates and 67 turkey isolates) were subjected to antimicrobial resistance profiling against 16 different antimicrobials that belong to ten different antibiotics classes (Table 3). Also 12 MRSA isolates (six isolates of each of the two positive chicken isolates) were subjected to antimicrobial resistance profiling against the same antimicrobials (Table 3). As shown in Table 3, the percentage of resistance of the 168 *S. aureus* isolates from poultry to the 16 tested antimicrobials were as follows: Ampicillin (94.6%), tetracycline (72%), penicillin (70.8%), doxycycline (62.5%), oxacillin with 2% NaCl (47.6%), azithromycin (44,6%), erythromycin (44,6%), vancomycin (36.8%), ciprofloxacin (32.1%), cefoxitin with 2% NaCl (31%), gentamicin (25.6%), kanamycin (26.8%), clindamycin (20.8%), rifampin (14.9%), trimethoprim/sulfamethazole (7.7%), chloramphenicol (5.4%). Also the percentage of resistance of the 12 MRSA isolates from poultry to the 16 tested antimicrobials were as follows: ampicillin (100%), penicillin (100%), cefoxitin with 2% NaCl (100%), oxacillin with 2% NaCl (100%), azithromycin (100%), erythromycin (100%), kanamycin (91.7%), trimethoprim/sulfamethazole (91.7%), clindamycin (58.3%), ciprofloxacin (33.3%), and no resistance reported for tetracycline, doxycyclin, vancomycin, gentamicin, rifampin, or chloramphenicol. As shown in Table 3, the percentage resistance to the 16 tested antimicrobials varied between chicken and turkey isolates. The percentage of resistance of the turkey isolates was higher than chicken isolates for all the antimicrobials tested except for clindamycin and trimethoprim/sulfamethoxazole.

A repeated measures MANOVA was used to test for a difference in antibiotic resistance profile among sample groups. Examined was the relative frequency of resistance to using antibiotics in Table 3, with chicken *S. aureus*, Chicken MRSA and turkey *S. aureus* being the ‘repeated’ samples. The logic here was that if the origin of the bacteria and/or drug resistance were the same then prevalence of resistance to a drug should be similarly high or low among groups. 

**Table 3:** Antimicrobial resistance of the *Staphylococcus aureus* and MRSA chicken and turkey isolates against 16 different antimicrobials.

Antimicrobial Resistance						
Antibiotic	Chicken		Turkey		Poultry	
	*S. aureus* * np/n (%)	MRSA np/n (%)	*S. aureus* np/n (%)	MRSA np/n (%)	*S. aureus* np/n (%)	*MRSA* np/n (%)
azithromycin	33/101 (32.7)	12/12 (100)	42/67 (62.7)	0/0 (0)	75/168 (44.6)	12/12 (100)
ciprofloxacin	22/101 (21.8)	4/12 (33.3)	32/67 (47.8)	0/0 (0)	54/168 (32.1)	4/12 (33.3)
gentamicin	20/101 (19.8)	12/12 (100)	23/67 (34.3)	0/0 (0)	43/168 (25.6)	0/12 (0)
oxacillin	22/101 (21.8)	12/12 (100)	58/67 (86.6)	0/0 (0)	80/168 (47.6)	12/12 (100)
cefoxitin	15/101 (14.9)	12/12 (100)	37/67 (55.2)	0/0 (0)	52/168 (31.0)	12/12 (100)
tetracycline	54/101 (53.5)	0/12 (0)	67/67 (100)	0/0 (0)	121/168 (72.0)	0/12 (0)
vancomycin	26/101 (25.7)	0/12 (0)	35/67 (52.2)	0/0 (0)	61/168 (36.3)	0/12 (0)
doxycycline	43/101 (42.6)	0/12 (0)	62/67 (92.5)	0/0 (0)	105/168 (62.5)	0/12 (0)
trimethoprim/sulfamethoxazole	8/101 (7.9)	11/12 (91.7)	5/67 (7.5)	0/0 (0)	13/168 (7.7)	11/12 (91.7)
clindamycin	29/101 (28.7)	7/12 (58.3)	6/67 (8.9)	0/0 (0)	35/168 (20.8)	7/12 (58.3)
penicillin	53/101 (52.5)	12/12 (100)	66/67 (98.5)	0/0 (0)	119/168 (70.8)	12/12 (100)
ampicillin	92/101 (91.1)	12/12 (100)	67/67 (100)	0/0 (0)	159/168 (94.6)	12/12 (100)
kanamycin	26/101 (25.7)	11/12 (91.7)	19/67 (28.4)	0/0 (0)	45/168 (26.8)	11/12 (91.7)
erythromycin	33/101 (32.7)	12/12 (100)	42/67 (62.7)	0/0 (0)	75/168 (44.6)	12/12 (100)
rifampin	10/101 (9.9)	0/12 (0)	15/67 (22.4)	0/0 (0)	25/168 (14.9)	0/12 (0)
chloramphenicol	3/101 (2.97)	0/12 (0)	6/67 (8.9)	0/0 (0)	9/168 (5.4)	0/12 (0)

* np: Number of positive isolates, n: Number of isolates collected.

Data tested were the arcsine square-root of the resistance relative frequency for each antibiotic in each of the test groups (chicken *S. aureus*, chicken MRSA, turkey *S. aureus*). A significant difference was observed among the three groups (F = 15.859, df = 2.14, *p* = 0.0003). We when went on to perform the same analysis just using chicken and turkey *S. aureus* since their profiles were most similar and they are the same organism. A significant difference was observed (F = 15.859, df = 1.15, *p* = 0.0006). What this says is the drug resistance profile differed among chicken *S. aureus*, chicken MRSA and turkey *S. aureus*.

The distribution of Multidrug Resistance (MDR) among the 168 *S. aureus* poultry isolates was as follows: 56 isolates resistant to one to four antimicrobials, 50 isolates resistant to five to seven antimicrobials, and 62 isolates resistant to more than seven antimicrobials. The distribution of Multidrug Resistance (MDR) among the 12 MRSA isolates was as follows: zero isolates resistant to one to four antimicrobials, one isolate resistant to five to seven antimicrobials, and 11 isolates resistant to more than seven antimicrobials. 64% (43/67) of the turkey isolates and 92% (11/12) of the MRSA isolates were highly multidrug resistant, being resistant to more than seven antimicrobials while only 19% (19/101) of the chicken isolates were resistant to seven antimicrobials. Multidrug resistance of *S. aureus* to three or more antimicrobial classes was 79% in turkey and 26% in chicken [30]. More ground turkey isolates were multidrug resistant than the beef ones [9].

Our poultry isolates were highly resistant to ampicillin (94.6%), tetracycline (72%), and penicillin (70.8%) (Table 3). Resistance to penicillin, ampicillin and tetracycline was also previously reported in poultry and was higher in turkey than chicken isolates [30]. In a previous study by Gundoga *et al.* in 2005, the highest resistance in chicken isolates was in penicillin (53.8%) [16]. Pu *et al.* reported resistances to penicillin (71%), ampicillin (68%), tetracycline (67%) and erythromycin (30%) from retail pork and beef in Louisiana [31]. All of the 12 MRSA isolates in our study (isolated from chicken meat) were resistant to ampicillin, penicillin, cefoxitin with 2% NaCl, oxacillin with 2% NaCl, azithromycin, and kanamycin and were susceptible to tetracycline, doxycycline, vancomycin, gentamicin, rifampin, and chloramphenicol.

In our study, more than 55% of the turkey isolates and more than 15% of the chicken isolates showed resistance to cefoxitin and/or oxacillin (Table 3). *S. aureus* recovered strains that showed resistance to cefoxitin and/or oxacillin were subjected to additional PCR protocols to check for the presence of the *mecC* or any *mecA* homologues. None of these isolates showed the presence of *mecA* gene or *mecC*. Phenotypic MRSA isolates not harboring the *mecA* gene were previously reported [9,30,32]. This might be due to over production of β-lactamase enzymes or the presence of a variant *mecA* gene that does not amplify with the available PCR primers. Whole genome sequencing might help identify new *mecA* homologues.

### 3.3. Toxin Genes Possession Screening of the Recovered Isolates

A total of 168 *Staphylococcus aureus* isolates (101 chicken isolates and 67 turkey isolates) were screened for 18 different toxin genes that belong to six different toxin gene groups (Table 4). Also a total of 12 MRSA isolates (six isolates of each of the two positive chicken isolates) were screened for the same 18 toxin genes (Table 4). As shown in Table 4, the prevalence of toxin genes in the 168 *S. aureus* isolates from poultry to the 18 tested toxin genes were as follows: *Hld* (76.2%), *hla* (75.6%), *hlb* (41.7%), lukE-*luk*D (31.5%), *sei* (24.4%), *seg* (16.7%), *tst* (5.4%), *lukS-lukF* (1.8%), *sea* (1.2%), *seb-sec* (1.2%), *see* (1.2%), *seh* (0.6%), *sec* (0.6%), *lukM* (0.6%), *sej* (0%), *sed* (0%), *eta* (0%), and *etb* (0%). Also the prevalence of toxin genes in the 12 chicken MRSA isolates to the eighteen tested toxin genes were as follows: *Hld* (100%), *hla* (100%), *lukE-luk*D (100%), *lukS-lukF* (66.7%), *hlb* (0%), *sei* (0%), *seg* (0%), *tst* (0%), *sea* (0%), *seb-sec* (0%), *see* (0%) *seh* (0%), *sec* (0%), *lukM* (0%), *sej* (0%), *sed* (0%), *eta* (0%), and *etb* (0%). As shown in Table 4, *S. aureus* hemolysin *hla* and *hld* genes were present at a higher percentage in chicken isolates than in the turkey ones, while hemolysin gene *hlb* was present slightly higher in turkey. The prevalence of enterotoxins *seg* (26.7%) and *sei* (39.6%) was higher in chicken than turkey isolates. Also no isolate of turkey harbored the enterotoxin genes *seb-sec, sea, sed, see*, *seh, sej,* the toxic shock syndrome toxin 1 gene *tst*, the exfloliative toxin genes *eta* or *etb*, and Leucocidin gene *lukM* or *lukS-lukF*. Two isolates (2/101) of *S. aureus* in chicken were positive for the enterotoxin genes *sea*, *sep-sec*, or *sep-sec*, while no isolates in turkey was positive for these entoretoxin genes. Nine isolates (9/101) of *S. aureus* in chicken were positive for the toxic shock syndrome toxin 1 gene *tst*, while no isolates in turkey was positive for this particular gene. Three isolates (3/101) of *S. aureus* in chicken were positive for the PVL gene *lukS-lukF* (3%), while no isolate from turkey was positive for this gene. As shown in Table 4, the percentage of the *lukE-luk*D gene in chicken isolates (46.5%) was higher than in the turkey ones (8.9%).

**Table 4:** Toxin gene screening of the *Staphylococcus aureus* and MRSA chicken and turkey isolates to 18 different toxin genes.

Prevalence of Toxin Gene						
Toxin Gene	Chicken		Turkey		Poultry	
	*S. aureus* * np/n (%)	MRSA np/n (%)	*S. aureus* np/n (%)	MRSA np/n(%)	*S. aureus* np/n (%)	MRSA np/n (%)
*sea*	2/101 (2)	0/12 (0)	0/67 (0)	0/0 (0)	2/168 (1.2)	0/12 (0)
*seb-sec*	2/101 (2)	0/12 (0)	0/67 (0)	0/0 (0)	2/168 (1.2)	0/12 (0)
*sec*	0/101 (0)	0/12 (0)	1/67 (1.5)	0/0 (0)	1/168 (0.6)	0/12 (0)
*sed*	0/101 (0)	0/12 (0)	0/67 (0)	0/0 (0)	0/168 (0)	0/12 (0)
*see*	2/101 (2)	0/12 (0)	0/67 (0)	0/0 (0)	2/168 (1.2)	0/12 (0)
*seg*	27/101 (26.7)	0/12 (0)	1/67 (1.5)	0/0 (0)	28/168 (16.7)	0/12 (0)
*seh*	1/101 (1)	0/12 (0)	0/67 (0)	0/0 (0)	1/168 (0.6)	0/12 (0)
*sei*	40/101 (39.6)	0/12 (0)	1/67 (1.5)	0/0 (0)	41/168 (24.4)	0/12 (0)
*sej*	0/101 (0)	0/12 (0)	0/67 (0)	0/0 (0)	0/168 (0)	0/12 (0)
*tst*	9/101 (8.9)	0/12 (0)	0/67 (0)	0/0 (0)	9/168 (5.4)	0/12 (0)
*eta*	0/101 (0)	0/12 (0)	0/67 (0)	0/0 (0)	0/168 (0)	0/12 (0)
*etb*	0/101 (0)	0/12 (0)	0/67 (0)	0/0 (0)	0/168 (0)	0/12 (0)
*lukE-lukD*	47/101 (46.5)	12/12 (100)	6/67 (8.9)	0/0 (0)	53/168 (31.5)	12/12 (100)
*lukM*	1/101 (1)	0/12 (0)	0/67 (0)	0/0 (0)	1/168 (0.6)	0/12 (0)
*hla*	82/101 (81.2)	12/12(100)	45/67 (67.2)	0/0 (0)	127/168 (75.6)	12/12 (100)
*hlb*	39/101 (38.6)	0/12 (0)	31/67 (46.3)	0/0 (0)	70/168 (41.7)	0/12 (0)
*hld*	83/101 (82.2)	12/12 (100)	45/67 (67.2)	0/0 (0)	128/168 (76.2)	12/12 (100)
*luks-lukF*	3/101 (3)	8/12 (66.7)	0/67 (0)	0/0 (0)	3/168 (1.8)	8/12 (66.7)

* np: Number of positive isolates, n: Number of isolates collected.

Repeated measures MANOVA statistical treatment was also used to test for toxin gene relative-frequency profile difference among chicken *S. aureus*, chicken MRSA and turkey *S. aureus* groups. A significant difference was observed among the three groups (F = 5.499, df = 2.16, *p* = 0.0152). We when went on to perform the same analysis just using chicken and turkey *S. aureus* since their profiles were most similar and they are the same organism. A significant difference was observed (F = 11.672, df = 1.17, *p* = 0.0033). Thus, toxin profile differed among chicken *S. aureus*, chicken MRSA and turkey *S. aureus*.

Most of our poultry isolates were positive for *hld* (76.2%), *hla* (75.6%), *hlb* (41.7%), *lukE*-*luk*D (31.5%), *sei* (24.4%), *seg* (16.7%), *tst* (5.4%), *lukS-lukF* (1.8%), *sea* (1.2%), *seb-sec* (1.2%), *see* (1.2%) *seh* (0.6%), *sec* (0.6%), *lukM* (0.6%), and no isolates were positive for *sej*, *sed*, *eta*, and *etb*. A study conducted to characterize *S. aureus* and MRSA isolated from Louisiana retail pork and beef meats for the possession of toxin genes showed that the most prevalent was *seg* and *sei* followed by *seh*, *sed*, *sej*, and *sea* while no isolates harbored *seb*, *sec* or *see* [31]. Another study in Italy reported that the prevalence of enterotoxin genes for *S. aureus* was 58.8% in meat and dairy products [3]. Therefore, as was the case in the prevalence and antimicrobial resistance data, toxin gene possession can also vary by meat type, processing facility, and geographic location.

## 4. Conclusions

The prevalence of *S. aureus* in retail turkey meat was higher than in the chicken meat. Prevalence did not vary much between conventional and organic chicken samples. Two chicken samples were positive for MRSA where one of them was from an organic source. PFGE identified the two MRSA strains as belonging to PFGE type USA 300 and USA 500 which are community acquired CA-MRSA suggesting a human based source of contamination. Multidrug resistance and the percentage of resistance to most of the antimicrobials tested were higher in the turkey isolates compared to the chicken ones. The hemolysin *hla* and *hld* genes, enterotoxins *seg* and *sei,* and leucocidins *lukE-lukD* were more prevalent in the chicken isolates. The PVL gene *lukS-lukF* was detected only in chicken and MRSA isolates. Despite MRSA being present at a very low prevalence in retail poultry in this study, *S. aureus* was highly prevalent and some of the recovered isolates were multidrug resistant to several antimicrobials including cefoxitin and possessed several toxin genes that are known to contribute to the virulence of this important foodborne bacterium.
